# Diagnostic testing for chest pain in a pediatric emergency department and rates of cardiac disease before and during the COVID-19 pandemic: a retrospective study

**DOI:** 10.3389/fped.2024.1366953

**Published:** 2024-04-30

**Authors:** Ayhan Atmanli, Kenneth Yen, Amy Z. Zhou

**Affiliations:** Division of Pediatric Emergency Medicine, Department of Pediatrics, University of Texas Southwestern Medical Center, Dallas, TX, United States

**Keywords:** chest pain, diagnostic testing, COVID-19, MIS-C, cardiac disease

## Abstract

**Objectives:**

Chest pain is a common chief complaint in pediatric emergency departments (EDs). Coronavirus disease-2019 (COVID-19) has been shown to increase the risk of cardiac disease. It remains unclear how COVID-19 changed how pediatric emergency clinicians approach patients presenting with chest pain. The goal of this study was to characterize the diagnostic testing for chest pain in a pediatric ED before and during the COVID-19 pandemic.

**Methods:**

This was a retrospective study of children between the ages of 2–17 years presenting to a pediatric ED from 1/1/2018–2/29/2020 (Pre-COVID-19) and 3/1/2020–4/30/2022 (COVID-19) with chest pain. We excluded patients with a previous history of cardiac disease.

**Results:**

Of the 10,721 encounters during the study period, 5,692 occurred before and 5,029 during COVID-19. Patient demographics showed minor differences by age, weight, race and ethnicity. ED encounters for chest pain consisted of an average of 18% more imaging studies during COVID-19, including 14% more EKGs and 11% more chest x-rays, with no difference in the number of echocardiograms. Compared to Pre-COVID-19, 100% more diagnostic tests were ordered during COVID-19, including cardiac markers Troponin I (*p* < 0.001) and BNP (*p* < 0.001). During COVID-19, 1.1% of patients had a cardiac etiology of chest pain compared with 0.7% before COVID-19 (*p* = 0.03).

**Conclusions:**

During COVID-19, pediatric patients with chest pain underwent more diagnostic testing compared to Pre-COVID-19. This may be due to higher patient acuity, emergence of multisystem inflammatory syndrome in children (MIS-C) that necessitated more extensive testing and possible changes in ED clinician behavior during COVID-19.

## 1 Introduction

Chest pain is a common chief complaint in pediatric emergency departments (EDs), accounting for 0.3%–2% of all ED visits (1, 2). In contrast to the adult population where cardiac etiologies are the most common cause of chest pain (3), pediatric chest pain is often due to benign, noncardiac etiologies (4). To differentiate benign from more serious etiologies, pediatric chest pain can be broadly categorized into noncardiac vs. cardiac. The reported incidence of chest pain of cardiac etiology presenting to pediatric EDs is highly variable, ranging from 0.6% to 12.6% of all ED visits for chest pain (1, 2, 5, 6).

Although pediatric chest pain typically has benign causes and prognoses, ED diagnostic testing for chest pain can lead to significant utilization of resources and healthcare costs, including consults and referrals to cardiology, and unnecessary testing (7, 8). The lack of consistency in the diagnostic approach is thought to be a contributing factor to this phenomenon (7, 9). As a result, there have been concerted efforts to implement standardized clinical evaluation pathways to reduce unnecessary resource utilization, thereby decreasing practice variation and healthcare costs (2, 7–10). The goal of standardized diagnostic approaches is to rule out serious cardiac etiologies of chest pain based on the history, physical exam and an electrocardiogram (EKG) so as to reserve more extensive testing for patients more likely to have a cardiac etiology.

With the outbreak of the novel severe acute respiratory syndrome coronavirus 2 (SARS-CoV-2), a global pandemic due to coronavirus disease-2019 (COVID-19) was declared in March 2020. As of May 2023, more than 15 million children in the U.S. have tested positive for COVID-19 (11). Acute infection with COVID-19 can cause myocarditis and arrhythmia and has been shown to confer a 15.7-fold increase in the risk for myocarditis compared to individuals who do not have COVID-19 (12). Although relatively rare, a post-infectious inflammatory syndrome known as multisystem inflammatory syndrome in children (MIS-C) develops in 0.5%–3.1% of children typically 3–6 weeks after infection with SARS-CoV-2 (13). MIS-C can cause myocarditis, coronary artery aneurysms and cardiovascular collapse (14). Similarly, myopericarditis post-mRNA COVID-19 vaccination is a recognized complication and tends to affect the pediatric population in the 14–18 years range, with the highest incidence among 12–40 year olds (15). Furthermore, it is estimated that up to 30% of patients with COVID-19 may continue to complain of chest pain months after acute infection (“long COVID”) (16). Taken together, the COVID-19 pandemic has added another layer of complexity to the differential diagnoses of chest pain in children.

It is unclear if and to what extent the COVID-19 pandemic has affected how pediatric emergency clinicians approach patients presenting with chest pain. We speculate that pediatric emergency clinicians have taken a more cautious approach in evaluating patients with chest pain during the COVID-19 pandemic with increased diagnostic testing. This study aims to characterize the extent of diagnostic workup for chest pain in a pediatric ED before and during the COVID-19 pandemic.

## 2 Methods

### 2.1 Data source

This was a retrospective study of children presenting to the pediatric EDs on the Children's Medical Center (CMC) campuses in Dallas, TX and Plano, TX with the chief complaint of chest pain. The CMC EDs have an annual volume of approximately 120,000 patient visits a year. Clinical pathways for pediatric chest pain did not exist at our institution in either of the two emergency departments during the study period. Data was abstracted from electronic medical records (Epic Systems Corporation, Verone, WI). The University of Texas Southwestern Medical Center Institutional Review Board exempted this work as non-human subject research.

### 2.2 Study design

We included pediatric patients aged 2–17 years who presented with the chief complaint of chest pain and excluded patients with a history of cardiac disease using International Classification of Diseases, Tenth Revision (ICD-10) codes (Supplementary Table S1). We abstracted data from ED encounters between 1 January 2018 and 30 April 2022. We defined the Pre-COVID-19 period as 1 January 2018–29 February 2020 and the COVID-19 period as 1 March 2020–30 April 2022. Data obtained were sex, age, weight, race, ethnicity, ED length of visit, disposition, chief complaints, imaging studies [EKG, chest x-ray (CXR), echocardiogram, point-of-care ultrasound (POCUS) echocardiogram, computed tomography (CT) of the chest, and others], laboratory tests, cardiology consults, cardiology outpatient referrals and ICD-10 codes of final diagnoses.

### 2.3 Relationship of cardiac diagnoses with COVID-19 cases in the U.S.

Cardiac diagnoses were identified using ICD-10 codes (Supplementary Table S1). The timing of cardiac diagnoses during COVID-19 was visually represented in relationship to the weekly number of COVID-19 cases in the U.S. Data on weekly COVID-19 cases were downloaded from the Centers for Disease Control (CDC): https://covid.cdc.gov/covid-data-tracker/#trends_weeklycases_select_00.

### 2.4 Data analysis

Continuous variables are reported as mean ± standard deviation and were tested for normality using Kolmogorov–Smirnov test. Normally distributed data were analyzed using the Student's *t*-test and data not following normal distribution were analyzed using Mann–Whitney test. Categorical variables are presented as numbers and percentages and were compared using *χ*^2^ test to compute odds ratios (ORs) with confidence intervals (CIs) and *p*-values. ORs are presented with the Pre-COVID-19 group as the reference. All tests were two-tailed. Data analysis was performed using Microsoft Excel (Microsoft Corporation, Redmond, WA; Version 2301) and Graphpad Prism software (Dotmatics, Boston, MA; Version 8.4.3). Statistical significance was defined as *p* < 0.05.

## 3 Results

### 3.1 Pediatric ED visits for chest pain

**Figure 1:**
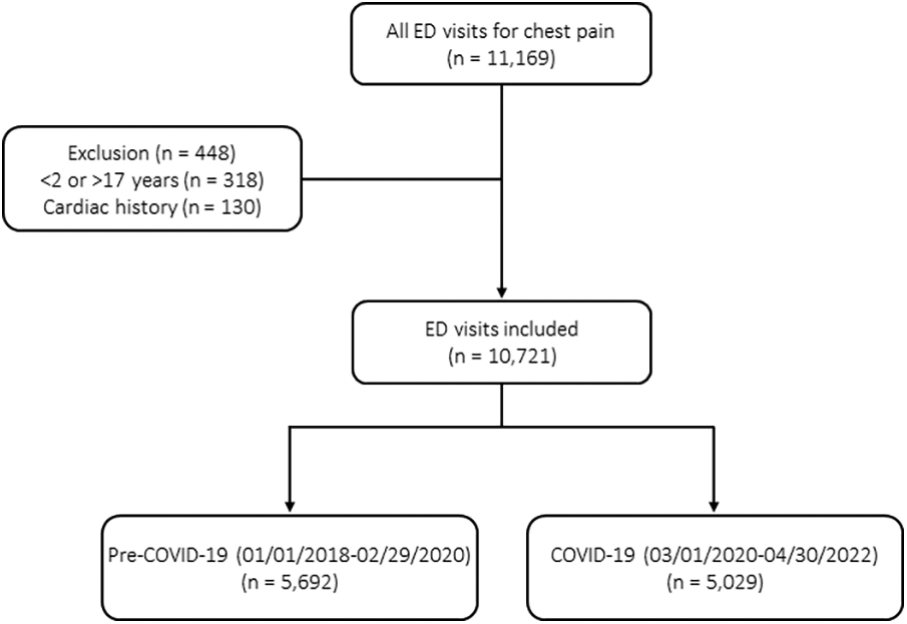
Flowchart of patient selection.

**Figure 2:**
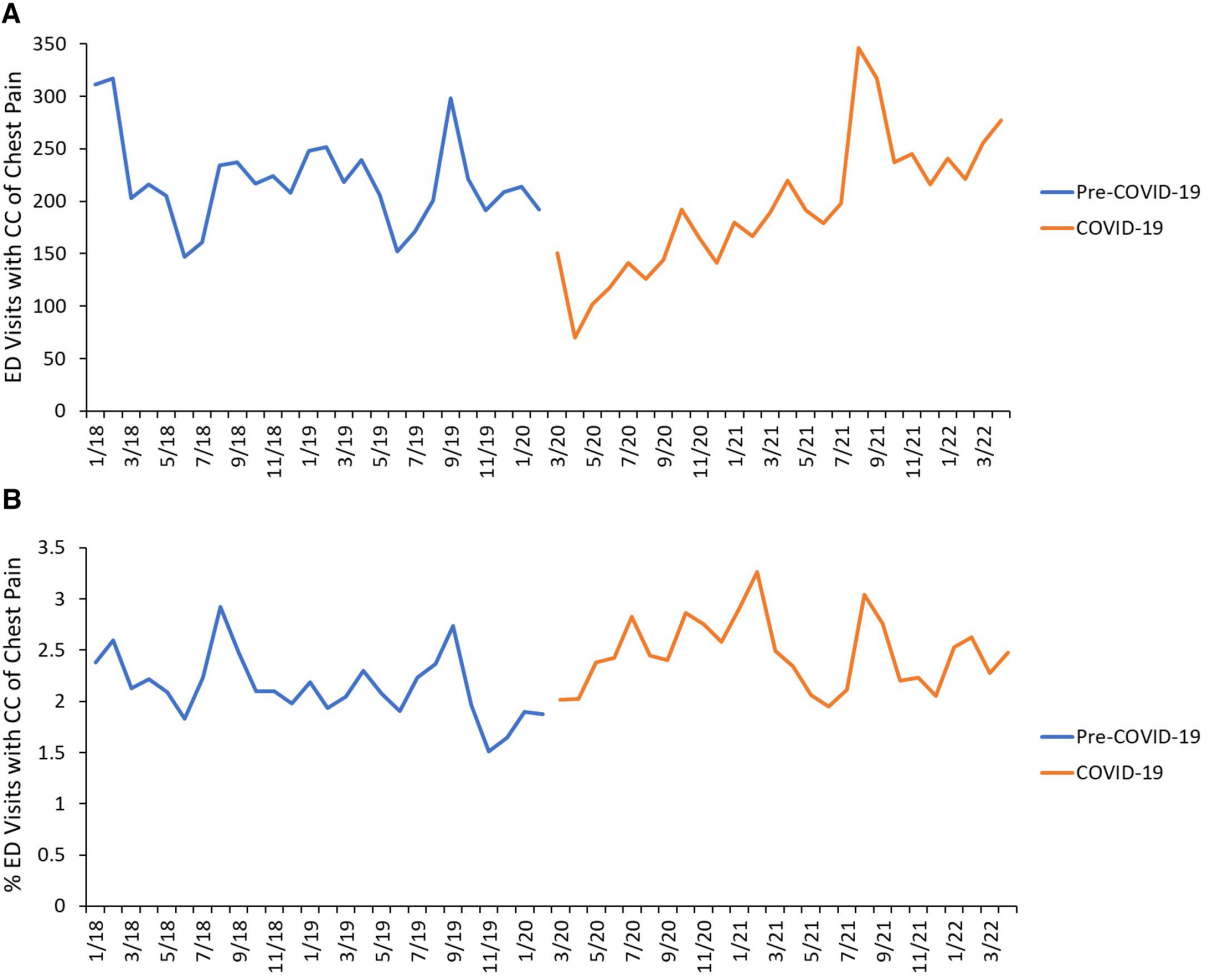
Timeline of ED visits. (**A**) Absolute numbers of ED visits for CC of chest pain during the study period, color-coded by study period. (**B**) Relative frequencies of ED visits for CC of chest pain during the study period, color-coded by study period. The Pre-COVID-19 period was defined as 1 January 2018–29 February 2020 and the COVID-19 period was defined as 1 March 2020–30 April 2022.

There were a total of 473,321 ED visits during the study period of which 11,169 visits presented with a chief complaint of chest pain. Of those, 10,721 visits met the inclusion criteria. There were 5,692 ED visits during Pre-COVID-19 and 5,029 ED visits during COVID-19 for chest pain (Figure 1). During COVID-19, the absolute number of ED visits for chest pain significantly decreased at the onset of the pandemic, while the relative number of ED visits for chest pain remained stable, then steadily increased throughout the year (Figure 2). ED visit trends for chest pain in 2021 were overall similar to pre-pandemic years. While the absolute number of ED visits for chest pain was similar between Pre-COVID-19 and COVID-19 (218 vs. 193 ED visits for chest pain per month, *p* = 0.10), there was a significant increase in the relative number of ED visits for chest pain during COVID-19 (2.1% vs. 2.5%, *p* = 0.001).

To evaluate for differences in the characteristics of patients who visited the ED before and during the COVID-19 pandemic, we analyzed patient demographics, and found small differences by age, weight and visit length (Table 1). There were no significant differences by race, ethnicity and disposition of patients.

**Table 1:** Patient demographics of ED visits for CC of chest pain before and during the COVID-19 pandemic.

	All encounters	Pre-COVID-19	COVID-19	*p*-value
Number of encounters with chest pain, *n* (%)	10,721 (100)	5,692 (53.1)	5,029 (46.9)	
Age (years), mean ± SD	12 ± 3.8	12 ± 3.8	12 ± 3.9	<0.001
Weight (kg), mean ± SD	54 ± 25.2	53 ± 24.3	56 ± 26.2	<0.001
Visit length (hours), mean ± SD	4.1 ± 3.4	4.0 ± 2.1	4.2 ± 4.4	<0.001
Sex				
Male, *n* (%)	5,098 (47.6)	2,695 (47.3)	2,403 (47.8)	0.744
Female, *n* (%)	5,623 (52.4)	2,997 (52.7)	2,626 (52.2)	0.756

Other disposition includes eloped, left without being seen, left against medical advice, transfer to another facility and transfer to operating room. ICU, intensive care unit.

### 3.2 Diagnostic testing for pediatric chest pain

We then evaluated for differences in the diagnostic testing during ED visits for chest pain before and during the pandemic. During the pandemic, 18.2% more imaging studies were obtained per ED visit compared to pre-pandemic (1.3 vs. 1.1 per ED visit, *p* < 0.001; Table 2). The odds of undergoing no imaging study were lower during the pandemic (OR, 0.7, 95% CI, 0.7–0.8, *p* < 0.001), while the odds of receiving two imaging studies was higher during the pandemic (OR, 1.4, 95% CI, 1.3–1.5, *p* < 0.001). The majority of patients before and during the pandemic received an EKG (57.7% vs. 65.7%) and/or CXR (53.6% vs. 59.6%), both of which were done more frequently during the pandemic (EKG, OR, 1.4, 95% CI, 1.3–3.0, *p* < 0.001 and CXR, OR, 1.3, 95% CI, 1.2–2.8, *p* < 0.001). While there was no significant difference in the number of echocardiograms ordered during the pandemic compared to pre-pandemic (OR, 1.6, 95% CI, 0.9–2.9, *p* = 0.18), 34 encounters during COVID-19 underwent POCUS echocardiography that was newly introduced in our ED during the pandemic.

**Table 2:** Rates of imaging studies for ED visits for chest pain before and during the COVID-19 pandemic.

	All encounters	Pre-COVID-19	COVID-19	% Change in proportion of ED visits	Odds ratio (95% CI)	*p*-value
Encounters with chest pain, *n* (%)	10,721 (100)	5,692 (53.1)	5,029 (46.9)			
Imaging studies, *n* (%)	12,751 (100)	6,375 (50.0)	6,376 (50.0)			
Imaging studies per encounter, mean ± SD	1.2 ± 0.8	1.1 ± 0.8	1.3 ± 0.8	18.2	N/A	<0.001
Encounters with no study, *n* (%)	2,728 (25.4)	1,618 (28.4)	1,110 (22.1)	−22.2	0.7 (0.7–0.8)	<0.001
Encounters with 1 study, *n* (%)	3,295 (30.7)	1,794 (31.5)	1,501 (29.8)	−5.4	0.9 (0.9–1.0)	0.06
Encounters with 2 studies, *n* (%)	4,638 (43.2)	2,259 (39.7)	2,379 (47.3)	19.1	1.4 (1.3–1.5)	<0.001
Encounters with ≥3 studies, *n* (%)	60 (0.6)	21 (0.4)	39 (0.8)	100.0	2.1 (1.3–3.6)	0.006
EKG, *n* (%)	6,589 (61.5)	3,287 (57.7)	3,302 (65.7)	13.9	1.4 (1.3–3.0)	<0.001
EKG only, *n* (%)	1,898 (17.7)	1,011 (17.8)	887 (17.6)	0.0	1.0 (0.9–1.1)	0.88
CXR, *n* (%)	6,051 (56.4)	3,053 (53.6)	2,998 (59.6)	11.2	1.3 (1.2–2.8)	<0.001
Echocardiography, *n* (%)	42 (0.4)	18 (0.3)	24 (0.5)	66.7	1.6 (0.9–2.9)	0.18
POCUS Echocardiography, *n* (%)	34 (0.3)	0 (0.0)	34 (0.7)	N/A	N/A	<0.001
CT Chest, *n* (%)	30 (0.3)	16 (0.3)	14 (0.3)	0.0	1.0 (0.5–2.0)	0.98
Other studies, *n* (%)	5 (0.1)	1 (0.0)	4 (0.1)	352.7	4.5 (0.8–55.5)	0.14

Odds Ratios are shown with the Pre-COVID-19 group as the reference. Point-of-care ultrasound (POCUS) echocardiography was not available during the Pre-COVID-19 period, thus Odds Ratio not applicable. EKG, electrocardiogram; CXR, chest x-Ray; CT, computed tomography.

We next analyzed the rates of diagnostic laboratory tests and found that during COVID-19 twice as many tests were obtained per ED visit compared to Pre-COVID-19 (1.8 vs. 0.9 tests per ED visit, *p* < 0.001). Rates of selected laboratory tests are shown in Supplementary Table S2. The number of encounters with at least one laboratory test was 42.4% during the pandemic and 26.2% pre-pandemic (OR, 2.1, 95% CI, 1.5–2.3, *p* < 0.001). During COVID-19, 11.2% and 3.9% of encounters underwent testing for cardiac markers Troponin I and brain natriuretic peptide (BNP), respectively, while the rates were 3.5% and 0.8%, respectively, before the pandemic (Troponin I, OR, 3.4, 95% CI, 2.9–4.1, *p* < 0.001; BNP, OR, 4.2, 95% CI, 3.7–7.2, *p* < 0.001). At least 16 (0.3%) encounters during COVID-19 received diagnostic testing for MIS-C as shown by the rates of tests for interleukin-6 and SARS-COV-2 IgG antibody. Lastly, during COVID-19, cardiology was consulted twice as often (1.8% vs. 0.9%, OR, 1.9, 95% CI, 1.4–2.7, *p* < 0.001) and referrals for outpatient cardiology follow-up five times more frequently (0.5% vs. 0.1%, OR, 4.1, 95% CI, 1.8–9.7, *p* < 0.001) compared to Pre-COVID-19. Taken together, our data demonstrate that during COVID-19 the diagnostic evaluation for pediatric chest pain included higher rates of diagnostic testing and cardiology involvement compared to Pre-COVID-19.

### 3.3 Cardiac etiologies of pediatric chest pain

We then analyzed for differences in cardiac diagnoses before and during the pandemic. The incidence of cardiac diagnoses was 0.9% (98 cases) during the study period; 0.7% Pre-COVID-19 (41 cases) and 1.1% during COVID-19 (57 cases; OR, 1.6, 95% CI, 1.1–2.4, *p* = 0.02; Table 3). The most common cardiac diagnosis was arrhythmia and accounted for 41.8% of all cardiac diagnoses; 35.1% during COVID-19 and 51.2% during Pre-COVID-19 (OR, 0.5, 95% CI, 0.2–1.2, *p* = 0.11). Although myocarditis was more frequent during COVID-19, the difference was not statistically significant (15.8% vs. 4.9%, OR, 3.7, 95% CI, 0.9–17.5, *p* = 0.09). There were 5 encounters with a diagnosis of MIS-C accounting for 8.8% of cardiac diagnoses during COVID-19.

**Table 3:** Rates of cardiac diagnoses of ED visits for chest pain before and during the COVID-19 pandemic.

	All encounters	Pre-COVID-19	COVID-19	% Change in proportion of cardiac diagnosis	Odds ratio (95% CI)	*p*-value
Encounters with chest pain, *n* (%)	10,721 (100)	5,692 (53.1)	5,029 (46.9)			
Encounters with cardiac diagnosis, *n* (%)	98 (0.9)	41 (0.7)	57 (1.1)	57.1	1.6 (1.1–2.4)	0.02
Diagnosis, *n* (% of cardiac diagnosis)						
Arrhythmia	41 (41.8)	21 (51.2)	20 (35.1)	−31.4	0.5 (0.2–1.2)	0.11
Pericardial disease	24 (24.5)	11 (26.8)	13 (22.8)	−14.9	0.8 (0.3–2.1)	0.65
Myocarditis	11 (11.2)	2 (4.9)	9 (15.8)	222.4	3.7 (0.9–17.5)	0.09
MIS-C	5 (5.1)	0 (0.0)	5 (8.8)	N/A	N/A	0.05
Unspecified	17 (17.3)	7 (17.1)	10 (17.5)	2.3	1.0 (0.4–2.7)	0.95

MIS-C, multisystem inflammatory syndrome in children.

Infection with SARS-COV-2 has been shown to increase the risk of cardiac disease (12, 13, 16). We analyzed the timing of cardiac diagnoses during COVID-19 in our study in correlation with the rates of weekly COVID-19 cases in the U.S. based on publicly available data from the CDC (Supplementary Figure S1). While we did not identify clear patterns of cardiac diagnoses, there was qualitatively a cluster of diagnoses of pericardial disease following the surge of COVID-19 cases in December 2020 and December 2021. Two of the five MIS-C diagnoses followed the surge of COVID-19 cases in December 2020 and another two followed the surge of cases in August 2021.

### 3.4 Diagnostic testing in patients with cardiac-related chest pain

We next analyzed the extent of diagnostic testing of encounters with a cardiac vs. non-cardiac diagnosis during the entire study period and found that encounters with a cardiac diagnosis received higher rates of imaging studies compared to non-cardiac diagnosis (1.9 vs. 1.2 studies per ED visit, *p* < 0.001, Supplementary Table S3). All encounters with a cardiac diagnosis underwent at least one imaging study. This is in contrast to non-cardiac diagnosis where only 74.3% of encounters underwent imaging studies. Encounters with a cardiac diagnosis received more laboratory tests compared to non-cardiac diagnosis (6.8 vs. 1.4 tests per ED visit, *p* < 0.001), including cardiac markers Troponin I (52.0% vs. 6.7%, OR, 15.1, 95% CI, 10.1–45.1, *p* < 0.001) and BNP (27.6% vs. 2.0%, OR, 18.7, 95% CI, 11.7–29.7, *p* < 0.001), and were more likely to receive at least one laboratory test (OR, 7.8, 95% CI, 4.8–12.6, *p* < 0.001). These data demonstrate that encounters with a cardiac diagnosis underwent more extensive diagnostic testing.

We next analyzed the rates of diagnostic testing in encounters with a cardiac diagnosis before and during the pandemic. Although the rates of imaging studies were higher during COVID-19 compared to Pre-COVID-19, the differences were not statistically significant (1.9 vs. 1.7 studies per ED visit, *p* = 0.08, Supplementary Table S4). Encounters during COVID-19 were less likely to receive an isolated EKG study without other tests (OR, 0.3, 95% CI, 0.1–0.8, *p* = 0.009). Rates of diagnostic laboratory tests were higher during COVID-19 than Pre-COVID-19 (8.7 vs. 4.1 tests per ED visit, *p* < 0.001). The number of encounters with at least one laboratory test was 87.7% during the pandemic and 68.3% pre-pandemic (OR, 3.3, 95% CI, 1.2–8.5, *p* < 0.001). During COVID-19, 59.6% and 36.8% of encounters underwent Troponin I and BNP studies, respectively, while the rates were 41.5% and 14.6%, respectively, before the pandemic (Troponin I, OR, 2.1, 95% CI, 0.9–4.7, *p* = 0.08; BNP, OR, 3.4, 95% CI, 1.2–9.4, *p* = 0.02). Taken together, our data suggest that encounters with a cardiac diagnosis underwent higher rates of laboratory testing, but similar rates of imaging studies during COVID-19 compared to Pre-COVID-19.

## 4 Discussion

We found that during COVID-19, compared to pre-pandemic (1) there was an increase in the percentage of ED visits for chest pain, (2) the diagnostic evaluation for chest pain included higher rates of testing and cardiology involvement (3) the incidence of cardiac diagnoses was greater and (4) encounters with a cardiac diagnosis received more diagnostic testing compared to encounters with a non-cardiac diagnosis.

We found that early during the pandemic the number of ED visits for chest pain declined sharply. This is in line with the decreased number of pediatric ED visits across the U.S. during the pandemic (17). Despite overall declines in ED visits, we found that the percentage of ED visits for chest pain increased during the pandemic, similar to findings that ED visits for certain types of injuries (e.g., drug poisonings, self-harm, and firearm injuries) and mental health complaints increased during the pandemic (17, 18). A large retrospective study did not find differences in the relative proportion of pediatric ED visits for chest pain during the pandemic, however, the study was limited to the first year of the pandemic (19). Non-cardiac chest pain in children has been shown to be associated with psychosocial distress, sleep problems, restriction of activities and anxiety symptoms (20, 21). It is possible that the increased mental health issues children experienced during the pandemic contributed to increased incidence of chest pain, especially since the majority of encounters included in our study had a non-cardiac diagnosis.Infection with SARS-CoV-2 has been shown to place children at risk for long term sequelae (“long COVID”), including persistent chest pain symptoms in up to 4.6% of patients with COVID-19 (22) lasting at least two months after acute infection (23), possibly contributing to ED visits for chest pain during the pandemic. However, it is also possible that a decreased number of ED visits during COVID-19, especially in the first year of the pandemic from March 2020 to March 2021, may have led to an increased percentage of ED visits for chest pain. We recognize that there various potential causes of the difference in the percentage of ED visits for chest pain between both study periods, which is beyond the scope of this discussion.

The number of imaging studies obtained during ED visits for pediatric chest pain varies widely in the literature, ranging from 16% (24) to 94% (6) for CXR, 20% (24) to 100% (25) for EKG and 1% (2, 5) to 8% (24) for echocardiograms. While the number of CXR and EKG obtained in our study was within the range reported in the literature, echocardiograms were less frequently obtained in our study. The frequency with which laboratory tests are obtained for pediatric chest pain ED visits also varies widely in the literature, from 5% (2) to 62% (6). The number of laboratory tests obtained in our study was within this range. Our study showed that patients with chest pain underwent more diagnostic testing during the pandemic. The reasons for these observations are manifold. First, it is possible that patients presented with higher acuity during the pandemic, warranting more extensive testing. Second, during times of high COVID-19 incidence, the index of suspicion for severe illness was likely higher, leading to more extensive testing. Third, the morbidity and mortality associated with COVID-19 likely changed the behavior of ED clinicians, such as their comfort level with making disposition decisions without diagnostic testing. It is well known that infectious disease outbreaks have the potential to cause significant psychological stress to physicians (26), which in turn may affect their clinical decision making. The increased diagnostic testing, however, was arguably appropriate and clinically indicated, given that they resulted in a higher number of cardiac diagnoses during COVID-19. Fourth, the diagnosis of MIS-C involves a plethora of laboratory tests, including cardiac biomarkers and potentially an echocardiogram (27), therefore the increased number of tests during COVID-19 was likely skewed by the diagnostic criteria for MIS-C. Fifth, certain tests such as procalcitonin, were not available prior to the pandemic. Technological advancement therefore represents an important aspect of how clinicians approach patients presenting to the ED. Lastly, a respiratory virus panel became an admission criterion during the pandemic, whereby the test had to be obtained even if it did not contribute to the diagnostic testing for chest pain.

We found that cardiac etiologies were more likely to be the underlying cause of chest pain in children during the pandemic than pre-pandemic. The incidence of cardiac-related chest pain in our study during both study periods was within previously reported range of 0.6%–12.6% (1, 2, 5, 6). Although statistically not significant, we found a three-fold higher incidence of myocarditis during COVID-19, suggesting that the pandemic may have been a contributing factor to the increased incidence. The incidence of pericardial disease was not different pre-pandemic compared to during the pandemic. Both myocarditis and pericarditis are predominantly caused by viruses via airborne transmission (28, 29). However, the prevalence of community respiratory viruses, including those that have been shown to cause both myocarditis and pericarditis, such as influenza viruses, adenovirus and enteroviruses, significantly declined at the onset of pandemic and increased to 50% of pre-pandemic prevalence only in Fall 2021 (30). The majority of myocarditis and pericarditis diagnoses in our study occurred prior to Fall 2021, suggesting that a viral etiology other than SARS-CoV-2 may have been unlikely, although this does not rule out other etiologies, such as bacterial infections and non-infectious causes. In our analysis, we found a cluster of pericardial disease diagnoses following the surge of COVID-19 cases in December 2020 and December 2021, suggesting a link to acute infection with SARS-CoV-2. However, we did not demonstrate a causative relationship between infection or vaccine and subsequent myocarditis or pericardial disease.

Taken together, our study showed that during COVID-19 pediatric patients visiting the ED for the chief complaint of chest pain received more diagnostic testing than pre-pandemic, which was followed by a proportional increase of the incidence of cardiac diagnoses. It is possible that the higher patient acuity seen in pediatric EDs during the COVID-19 pandemic (31, 32) resulted in higher testing rates and cardiac diagnoses. The higher acuity could be partly attributed to the effects of COVID-19 on the cardiovascular system through direct infection, post-infectious sequelae and vaccination (12–16). This was likely exacerbated by the pandemic causing delays medical care due to widespread outpatient clinic closures and fear of exposure to SARS-CoV-2 in healthcare settings (33).

Our study has several limitations. First, despite being one of the largest pediatric EDs in the country, our single-center results may not be generalizable to other patient populations across the U.S. or in other countries. Second, our large sample size may have contributed to statistical significance for differences that are clinically insignificant. Lastly, we cannot exclude the possibility that the clinicians' decision to order more testing during the COVID-19 pandemic was due to their prior expectation of cardiac disease given the relationship between COVID-19 and cardiac disease.

In conclusion, during COVID-19 the proportion of ED visits for chest pain was higher, more diagnostic testing was done, and the incidence of cardiac diagnoses was higher compared to the pre-pandemic period. This may be due to higher patient acuity during the pandemic and thus higher pre-test probability for cardiac disease, technological advancement leading to novel imaging tools and laboratory tests, and the emergence of MIS-C which necessitated more extensive testing when suspected. Alternatively, our data suggest a possible change in ED clinician behavior during the pandemic. Given the higher rate of cardiac diagnoses during COVID-19, the increased diagnostic testing may have been justified. Our data suggest that when the local prevalence of infectious diseases known to increase the risk of cardiac disease in children, such as COVID-19, is high, a reasonably intensified diagnostic assessment may be warranted to rule out life-threatening cardiac disease.

## Data Availability

The raw data supporting the conclusions of this article will be made available by the authors, without undue reservation.

## Supplementary material

Supplementary Table S1

ICD-10 codes for cardiac disease.

Supplementary Table S2

Rates of laboratory tests for ED visits for chest pain before and during the COVID-19 pandemic.

Supplementary Table S3

Rates of imaging studies for ED visits for chest pain with non-cardiac vs cardiac diagnosis during the entire study period.

Supplementary Table S4

Rates of imaging studies for ED visits for chest pain with cardiac diagnosis before and during the COVID-19 pandemic.

Supplementary Figure S1

Temporal plot of the incidence of cardiac diagnoses during COVID-19 and weekly COVID-19 cases in the U.S. Weekly numbers of COVID-19 cases in the U.S. were obtained from the website of the CDC (see Methods). Cardiac diagnoses made during the COVID-19 pandemic as shown in Table 3 were plotted according to the date of the respective ED encounter. Each dot represents one ED encounter and is color-coded by cardiac diagnosis.
