# Supplementary material for: Diagnostic testing for chest pain in a pediatric emergency department and rates of cardiac disease before and during the COVID-19 pandemic: a retrospective study
Source: Front Pediatr. 2024 Apr 30;12:1366953. doi: 10.3389/fped.2024.1366953 (PMC11091279; doi:10.3389/fped.2024.1366953)
Supplement: Supplementary file 1 [file Table1.docx]

**Supplementary Table 1. ICD-10 codes for cardiac disease.**

| **ICD-10 Code** | **Condition** |
| --- | --- |
| I05-I09 codes | Rheumatic heart disease |
| I20-I25 codes | Ischemic heart disease |
| I27 codes | Pulmonary hypertension |
| I30, I31, I32 codes | Pericarditis and other diseases of pericardium |
| I33, I38, I39 codes | Acute and subacute endocarditis |
| I34, I35, I36, I37 codes | Nonrheumatic mitral/aortic/tricuspid/pulmonary valve disorders |
| I40, I41 codes | Myocarditis |
| I42, I43 codes | Cardiomyopathy |
| I44, I45, I47, I48, I49 codes | Cardiac arrhythmia |
| I46 codes | Cardiac arrest |
| I50 codes | Heart failure |
| I51, I52 codes | Complications and ill-defined descriptions of heart disease and other heart conditions |
| I5A | Myocardial injury |
| M30.3 | Kawasaki disease |
| Q20, Q21, Q22, Q23, Q24, Q25 codes; Q26.0-Q26.4, Q26.8-Q26.9 | Structural anomalies of the heart and great arteries/veins |
| Q87.4 codes | Marfan syndrome |
| T86.2 + T86.3 | Complications of heart and heart-lung transplants |
| Z87.74 | Personal history of (corrected) congenital malformations of heart and circulatory system |
