# Supplementary material for: Diagnostic testing for chest pain in a pediatric emergency department and rates of cardiac disease before and during the COVID-19 pandemic: a retrospective study
Source: Front Pediatr. 2024 Apr 30;12:1366953. doi: 10.3389/fped.2024.1366953 (PMC11091279; doi:10.3389/fped.2024.1366953)
Supplement: Supplementary file 2 [file Table2.docx]

**Supplementary Table 2. Rates of laboratory tests for ED visits for chest pain before and during the COVID-19 pandemic.**

|  | **All Encounters** | **Pre-COVID-19** | **COVID-19** | **% Change in Proportion of ED Visits** | **Odds Ratio (95% CI)** | ***p* value** |
| --- | --- | --- | --- | --- | --- | --- |
| Encounters with chest pain, n (%) | 10,721 (100) | 5,692 (53.1) | 5,029 (46.9) |  |  |  |
| Tests, n (%) | 14,521 (100) | 5,309 (36.6) | 9,212 (63.4) |  |  |  |
| Tests per encounter, mean ± SD | 1.4 ± 2.8 | 0.9 ± 2.1 | 1.8 ± 3.4 | 100.0 | N/A | <0.001 |
| Encounters with ≥1 test, n (%) | 3,624 (33.8) | 1,491 (26.2) | 2,133 (42.4) | 61.8 | 2.1 (1.5-2.3) | <0.001 |
|  |  |  |  |  |  |  |
| CBC + Differential, n (%) | 1,887 (17.6) | 811 (14.2) | 1,076 (21.4) | 50.7 | 1.6 (1.5-1.8) | <0.001 |
| BMP, n (%) | 580 (5.4) | 310 (5.4) | 270 (5.4) | 0.0 | 1.0 (0.8-1.2) | 0.86 |
| CMP, n (%) | 1,193 (11.1) | 430 (7.6) | 763 (15.2) | 100.0 | 2.2 (1.9-2.5) | <0.001 |
| RFP, n (%) | 25 (0.2) | 11 (0.2) | 14 (0.3) | 50.0 | 1.4 (0.7-3.3) | 0.42 |
| Electrolytes with gap, n (%) | 16 (0.2) | 10 (0.2) | 6 (0.1) | -50.0 | 0.7 (0.3-1.9) | 0.62 |
| Ionized Calcium, n (%) | 137 (1.3) | 61 (1.1) | 76 (1.5) | 36.4 | 1.4 (1.0-2.0) | 0.04 |
| Magnesium, n (%) | 279 (2.6) | 122 (2.1) | 157 (3.1) | 47.6 | 1.5 (1.2-1.9) | 0.002 |
| Phosphorus, n (%) | 261 (2.4) | 115 (2.0) | 146 (2.9) | 45.0 | 1.5 (1.1-1.9) | 0.003 |
| Blood gas, n (%) | 209 (1.9) | 92 (1.6) | 117 (2.3) | 43.8 | 1.5 (1.1-1.9) | 0.01 |
| AST, n (%) | 52 (0.5) | 20 (0.4) | 32 (0.6) | 50.0 | 1.8 (1.1-3.1) | 0.04 |
| ALT, n (%) | 51 (0.5) | 19 (0.3) | 32 (0.6) | 100.0 | 1.9 (1.1-3.4) | 0.02 |
| GGT, n (%) | 117 (1.1) | 53 (0.9) | 64 (1.3) | 44.4 | 1.4 (1.0-2.0) | 0.09 |
| Ammonia, n (%) | 24 (0.2) | 5 (0.1) | 19 (0.4) | 300.0 | 4.3 (1.7-10.6) | 0.002 |
| ALP, n (%) | 38 (0.4) | 14 (0.3) | 24 (0.5) | 66.7 | 2.0 (1.0-3.7) | 0.05 |
| Total and direct Bilirubin, n (%) | 64 (0.6) | 23 (0.4) | 41 (0.8) | 100.0 | 2.0 (1.2-3.5) | 0.008 |
| Albumin, n (%) | 33 (0.3) | 9 (0.2) | 24 (0.5) | 150.0 | 3.0 (1.5-6.5) | 0.004 |
| LDH, n (%) | 92 (0.9) | 27 (0.5) | 65 (1.3) | 130.0 | 2.8 (1.8-4.3) | <0.001 |
| Triglycerides, n (%) | 19 (0.2) | 1 (0.0) | 18 (0.4) | 2185.7 | 20.4 (3.7-214.4) | <0.001 |
| PT w/ INR, n (%) | 212 (2.0) | 83 (1.5) | 129 (2.6) | 73.3 | 1.8 (1.4-2.4) | <0.001 |
| APT, n (%) | 190 (1.8) | 73 (1.3) | 117 (2.3) | 76.9 | 1.8 (1.4-2.5) | <0.001 |
| TSH, n (%) | 332 (3.1) | 141 (2.5) | 191 (3.8) | 52.0 | 1.6 (1.3-1.9) | <0.001 |
| T4, n (%) | 263 (2.5) | 107 (1.9) | 156 (3.1) | 63.2 | 1.7 (1.3-2.1) | <0.001 |
| CRP, n (%) | 584 (5.4) | 189 (3.3) | 395 (7.9) | 140.0 | 2.5 (2.1-3.0) | <0.001 |
| ESR, n (%) | 438 (4.1) | 140 (2.5) | 298 (5.9) | 136.0 | 2.5 (2.0-3.1) | <0.001 |
| Procalcitonin, n (%) | 166 (1.5) | 0 (0.0) | 166 (3.3) | N/A | N/A | <0.001 |
| Ferritin, n (%) | 81 (0.8) | 13 (0.2) | 68 (1.4) | 600.0 | 6.0 (3.4-10.6) | <0.001 |
| Interleukin-6, n (%) | 16 (0.1) | 0 (0.0) | 16 (0.3) | N/A | N/A | <0.001 |
| D-Dimer, n (%) | 304 (2.8) | 87 (1.5) | 217 (4.3) | 186.7 | 2.9 (2.3-3.8) | <0.001 |
| Fibrinogen, n (%) | 85 (0.8) | 10 (0.2) | 75 (1.5) | 650.0 | 8.6 (4.6-17.2) | <0.001 |
| Troponin I, n (%) | 765 (7.1) | 202 (3.5) | 563 (11.2) | 220.0 | 3.4 (2.9-4.1) | <0.001 |
| BNP, n (%) | 239 (2.2) | 44 (0.8) | 195 (3.9) | 387.5 | 4.2 (3.7-7.2) | <0.001 |
| CK, n (%) | 181 (1.7) | 63 (1.1) | 118 (2.3) | 109.1 | 2.2 (1.6-2.9) | <0.001 |
| CK-MB, n (%) | 27 (0.3) | 24 (0.4) | 3 (0.1) | -75.0 | 0.1 (0.0-0.4) | <0.001 |
| Myoglobin, n (%) | 2 (0.0) | 0 (0.0) | 2 (0.0) | N/A | N/A | 0.22 |
| SARS-COV-2 IgG Antibody, n (%) | 16 (0.2) | 0 (0.0) | 16 (0.3) | N/A | N/A | <0.001 |
| Respiratory Viral Panel, n (%) | 1,151 (10.7) | 97 (1.7) | 1,054 (21.0) | 1135.3 | 15.3 (12.4-18.9) | <0.001 |
| COVID-19 PCR, n (%) | 411 (3.8) | 0 (0.0) | 411 (8.2) | N/A | N/A | <0.001 |
| COVID-19 Antigen Assay, n (%) | 167 (1.6) | 0 (0.0) | 167 (3.3) | N/A | N/A | <0.001 |
| Blood Culture, n (%) | 295 (2.8) | 131 (2.3) | 164 (3.3) | 43.5 | 1.4 (1.1-1.8) | 0.003 |
| Urinalysis, n (%) | 1,347 (12.6) | 677 (11.9) | 670 (13.3) | 11.8 | 1.1 (1.0-1.3) | 0.03 |
| Urine Culture, n (%) | 871 (8.1) | 434 (7.6) | 436 (8.7) | 14.5 | 1.2 (1.0-1.3) | 0.05 |
| Urinalysis with urine culture, n (%) | 832 (7.8) | 407 (7.2) | 425 (8.5) | 18.1 | 1.2 (1.0-1.4) | 0.01 |
| Urine Drug Screen, n (%) | 469 (4.4) | 254 (4.5) | 215 (4.3) | -4.4 | 1.0 (0.8-1.2) | 0.67 |

Odds Ratios are shown with the Pre-COVID-19 group as the reference. Procalcitonin, SARS-CoV-2 and COVID-19 laboratory tests were not available during the Pre-COVID-19 period, thus Odds Ratio not applicable. CBC, complete blood count; BMP, basic metabolic panel; CMP, comprehensive metabolic panel; RFP, renal function panel; AST, aspartate transaminase; ALT, alanine transaminase; GGT, gamma-glutamyl transferase; ALP, alkaline phosphatase; LDH, lactate dehydrogenase; PT, prothrombin time; INR, international normalized ratio; APT, activated partial thromboplastin time; TSH, thyroid stimulating hormone; CRP, C-reactive protein; ESR, erythrocyte sedimentation rate; BNP, brain natriuretic peptide; CK, creatine kinase; CK-MB, creatine kinase MB fraction; SARS-CoV-2, severe acute respiratory syndrome coronavirus 2; COVID-19, coronavirus disease-2019.
