# Supplementary material for: Diagnostic testing for chest pain in a pediatric emergency department and rates of cardiac disease before and during the COVID-19 pandemic: a retrospective study
Source: Front Pediatr. 2024 Apr 30;12:1366953. doi: 10.3389/fped.2024.1366953 (PMC11091279; doi:10.3389/fped.2024.1366953)
Supplement: Supplementary file 3 [file Table3.docx]

**Supplementary Table 3. Rates of imaging studies for ED visits for chest pain with non-cardiac vs cardiac diagnosis during the entire study period.**

|  | **All Encounters** | **Non-Cardiac Diagnosis** | **Cardiac Diagnosis** | **% Change in Proportion of ED Visits** | **Odds Ratio (95% CI)** | ***p* value** |
| --- | --- | --- | --- | --- | --- | --- |
| Encounters with chest pain, n (%) | 10,721 (100) | 10,623 (99.1) | 98 (0.9) |  |  |  |
| Imaging studies, n (%) | 12,751 (100) | 12,569 (98.6) | 182 (1.4) |  |  |  |
| Imaging studies per encounter, mean ± SD | 1.2 ± 0.8 | 1.2 ± 0.8 | 1.9 ± 0.6 | 58.3 | N/A | <0.001 |
| Encounters with no study, n (%) | 2,728 (25.4) | 2,728 (25.7) | 0 (0.0) |  |  |  |
| Encounters with 1 study, n (%) | 3,295 (30.7) | 3,271 (30.8) | 24 (24.5) | -20.5 | 0.7 (0.5-1.1) | 0.18 |
| Encounters with 2 studies, n (%) | 4,638 (43.2) | 4,573 (43.0) | 65 (66.3) | 54.2 | 2.6 (1.7-4.0) | <0.001 |
| Encounters with ≥3 studies, n (%) | 60 (0.6) | 51 (0.5) | 9 (9.2) | 1740.0 | 21.0 (10.1-42.2) | <0.001 |
|  |  |  |  |  |  |  |
| EKG, n (%) | 6,589 (61.5) | 6,497 (61.2) | 92 (93.9) | 53.4 | 9.7 (4.4-20.7) | <0.001 |
| EKG only, n (%) | 1,898 (17.7) | 1,879 (17.7) | 19 (19.4) | 9.6 | 1.1 (0.5-1.8) | 0.66 |
| CXR, n (%) | 6,051 (56.4) | 5,981 (56.3) | 70 (71.4) | 26.8 | 1.9 (1.3-3.0) | 0.003 |
| Echocardiography, n (%) | 42 (0.4) | 25 (0.2) | 17 (17.3) | 8550.0 | 100.1 (51.0-193.4) | <0.001 |
| POCUS Echocardiography, n (%) | 34 (0.3) | 33 (0.3) | 1 (1.0) | 230.0 | 3.3 (0.3-19.1) | 0.21 |
| CT Chest, n (%) | 30 (0.3) | 29 (0.3) | 1 (1.0) | 230.0 | 3.8 (0.4-22.1) | 0.16 |
| Other studies, n (%) | 5 (0.1) | 4 (0.0) | 1 (1.0) | 2608.9 | 27.4 (2.2-166.3) | <0.001 |

Odds Ratios are shown with the non-cardiac diagnosis group as the reference. EKG, electrocardiogram; CXR, chest X-Ray; POCUS, point-of-care ultrasound; CT, computed tomography.
