# Supplementary material for: Diagnostic testing for chest pain in a pediatric emergency department and rates of cardiac disease before and during the COVID-19 pandemic: a retrospective study
Source: Front Pediatr. 2024 Apr 30;12:1366953. doi: 10.3389/fped.2024.1366953 (PMC11091279; doi:10.3389/fped.2024.1366953)
Supplement: Supplementary file 4 [file Table4.docx]

**Supplementary Table 4. Rates of imaging studies for ED visits for chest pain with cardiac diagnosis before and during the COVID-19 pandemic.**

|  | **All Encounters** | **Pre-COVID-19** | **COVID-19** | **% Change in Proportion of ED Visits** | **Odds Ratio (95% CI)** | ***p* value** |
| --- | --- | --- | --- | --- | --- | --- |
| Encounters with cardiac diagnosis, n (%) | 98 (100) | 41 (41.8) | 57 (58.2) |  |  |  |
| Imaging studies, n (%) | 182 (100) | 71 (39.0) | 111 (61.0) |  |  |  |
| Imaging studies per encounter, mean ± SD | 1.9 ± 0.6 | 1.7 ± 0.6 | 1.9 ± 0.6 | 11.7 | N/A | 0.08 |
| Encounters with no study, n (%) | 0 (0.0) | 0 (0.0) | 0 (0.0) |  |  |  |
| Encounters with 1 study, n (%) | 24 (24.5) | 14 (34.1) | 10 (17.5) | -48.7 | 0.4 (0.2-1.0) | 0.06 |
| Encounters with 2 studies, n (%) | 65 (66.3) | 24 (58.5) | 41 (71.9) | 22.9 | 1.8 (0.8-4.3) | 0.17 |
| Encounters with ≥3 studies, n (%) | 9 (9.2) | 3 (7.3) | 6 (10.5) | 43.8 | 1.5 (0.4-5.7) | 0.73 |
|  |  |  |  |  |  |  |
| EKG, n (%) | 92 (93.9) | 39 (95.1) | 53 (93.0) | -2.2 | 0.7 (0.1-3.1) | 0.66 |
| EKG only, n (%) | 19 (19.4) | 13 (31.7) | 6 (10.5) | -66.9 | 0.3 (0.1-0.8) | 0.009 |
| CXR, n (%) | 70 (71.4) | 27 (65.9) | 43 (75.4) | 14.4 | 1.6 (0.7-3.6) | 0.3 |
| Echocardiography, n (%) | 17 (17.3) | 4 (9.8) | 13 (22.8) | 132.7 | 2.7 (0.9-8.1) | 0.09 |
| POCUS Echocardiography, n (%) | 1 (1.0) | 0 (0.0) | 1 (1.8) |  |  |  |
| CT Chest, n (%) | 1 (1.0) | 1 (2.4) | 0 (0.0) |  |  |  |
| Other studies, n (%) | 1 (1.0) | 0 (0.0) | 1 (1.8) |  |  |  |

Odds Ratios are shown with the Pre-COVID-19 group as the reference. EKG, electrocardiogram; CXR, chest X-Ray; POCUS, point-of-care ultrasound; CT, computed tomography.
