# Supplementary figures and images for: Diagnostic testing for chest pain in a pediatric emergency department and rates of cardiac disease before and during the COVID-19 pandemic: a retrospective study
Source: Front Pediatr. 2024 Apr 30;12:1366953. doi: 10.3389/fped.2024.1366953 (PMC11091279; doi:10.3389/fped.2024.1366953)

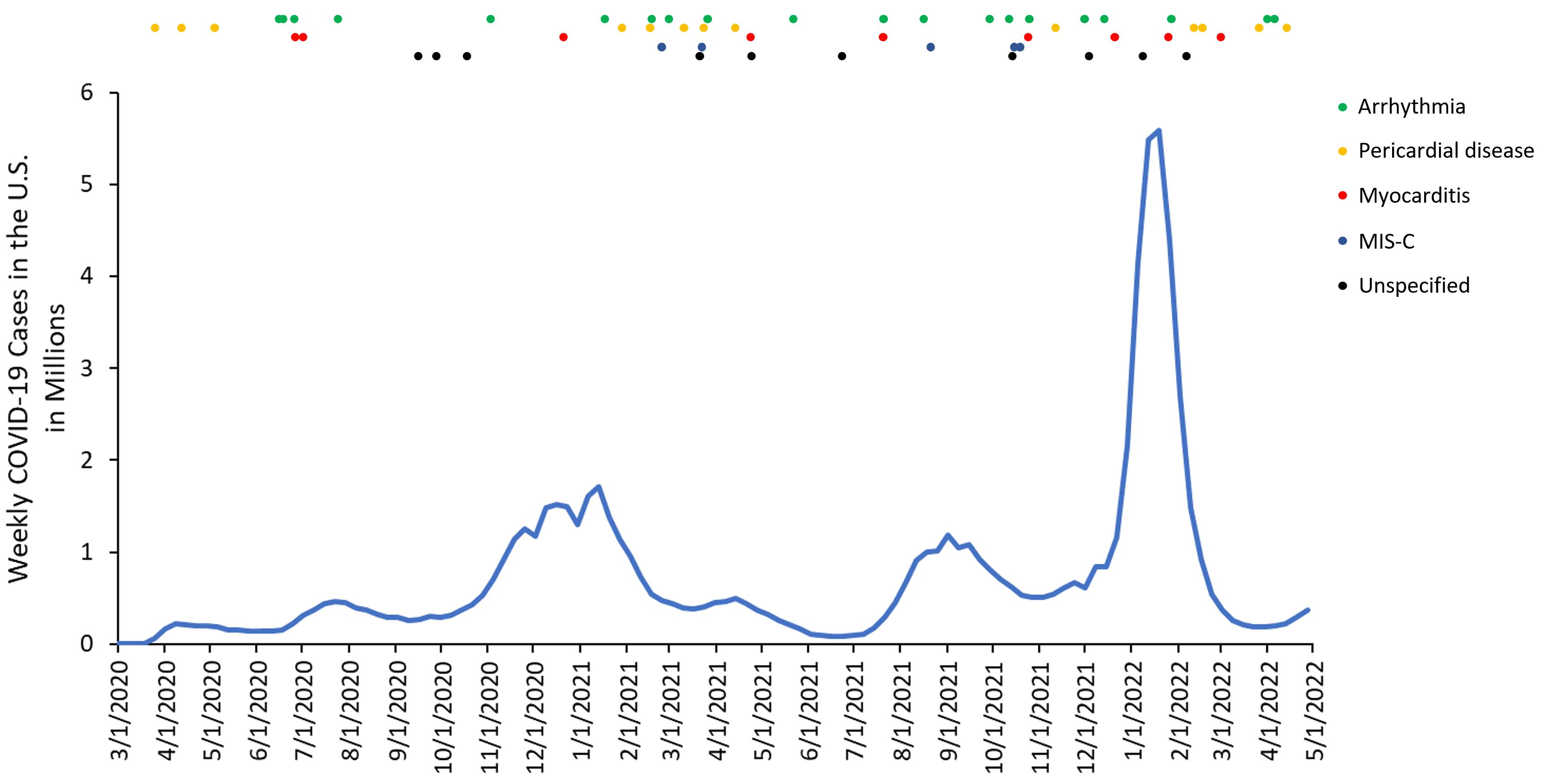

Supplement: Supplementary file 5 [file Image1.jpg]
